# Breed Locally, Disperse Globally: Fine-Scale Genetic Structure Despite Landscape-Scale Panmixia in a Fire-Specialist

**DOI:** 10.1371/journal.pone.0067248

**Published:** 2013-06-25

**Authors:** Jennifer C. Pierson, Fred W. Allendorf, Pierre Drapeau, Michael K. Schwartz

**Affiliations:** 1 Wildlife Biology Program, University of Montana, Missoula, Montana, United States of America; 2 Division of Biological Sciences, University of Montana, Missoula, Montana, United States of America; 3 Centre d’étude de la forêt (CEF), Département des sciences biologiques, Université du Québec à Montréal, Montréal, Québec, Canada; 4 United States Forest Service, Rocky Mountain Research Station, Missoula, Montana, United States of America; The Australian National University, Australia

## Abstract

An exciting advance in the understanding of metapopulation dynamics has been the investigation of how populations respond to ephemeral patches that go ‘extinct’ during the lifetime of an individual. Previous research has shown that this scenario leads to genetic homogenization across large spatial scales. However, little is known about fine-scale genetic structuring or how this changes over time in ephemeral patches. We predicted that species that specialize on ephemeral habitats will delay dispersal to exploit natal habitat patches while resources are plentiful and thus display fine-scale structure. To investigate this idea, we evaluated the effect of frequent colonization of ephemeral habitats on the fine-scale genetic structure of a fire specialist, the black-backed woodpecker (*Picoides arcticus*) and found a pattern of fine-scale genetic structure. We then tested for differences in spatial structure between sexes and detected a pattern consistent with male-biased dispersal. We also detected a temporal increase in relatedness among individuals within newly burned forest patches. Our results indicate that specialist species that outlive their ephemeral patches can accrue significant fine-scale spatial structure that does not necessarily affect spatial structure at larger scales. This highlights the importance of both spatial and temporal scale considerations in both sampling and data interpretation of molecular genetic results.

## Introduction

The genetic structure of metapopulations is driven by habitat quality within patches, which influences the demographic success of a species, and movement of individuals, or gene flow, among habitat patches [1]. Species adapted to early successional habitats have evolved with natural disturbance regimes that create dynamic spatial and temporal patterns of ephemeral habitat patches. Disturbance-dependent species may fit into a habitat-tracking metapopulation framework [2] where organisms track habitat through space and time [3], [4]. However, highly ephemeral habitats add a layer of complexity that leads to unclear predictions regarding the spatial genetic structure of disturbance-dependent species because the spatial context of habitat patches is constantly changing.

For a dynamic metapopulation, large-scale genetic structure will be the result of patterns of frequent colonization and extinction of patches and how that varies in space and time. Frequent colonization of habitat patches can increase or decrease the amount of divergence among occupied patches depending on the source of the propagules [5], [6]. Highly ephemeral habitats, such as burned forest, represent a situation where both the source and destination of colonists vary in space and time. Giles and Goudet [7] examined the genetic metapopulation structure of an early successional plant, *Silene dioca*, and found temporal variation in the amount of genetic divergence among populations, with generally ‘young’ and ‘old’ populations being more divergent than populations of an intermediate age. While other previous research has shown that rapid habitat turnover can result in genetic homogenization across large spatial scales [8].

Little is known about the effects of ephemeral habitat patches on fine-scale genetic structure, or on changes in fine-scale structure through time. Patterns of fine-scale genetic structure can result from an individual’s behavior within patches such as the presence of juveniles prior to natal dispersal, variation in reproductive success among individuals in the population, and general patterns of natal and breeding dispersal including sex-biased dispersal and dispersal distance [9]–[12].

In habitat-tracking metapopulations, once an area is colonized, fine-scale genetic structure can build during the time the habitat is occupied due to the presence of family groups, limited dispersal distance, and timing of natal dispersal by juveniles. Large-scale genetic structure tends to reflect the long-term genetic signatures of these individual behaviors. That is, over time, individuals that depend on natural disturbances to create habitat will colonize, occupy and leave patches for new ones and the result of this behavior at the large scale will be reflected in the large-scale genetic structure. Fine-scale genetic structure within these recently ‘created’ habitat patches will reflect more recent behaviors by individuals occupying the current patches such as the distance the individual travelled to colonize the patch and the dispersal behavior of their offspring. Dispersal is often sex-biased [13] and long-distance dispersal by only one sex can reduce the amount of genetic spatial structure detected despite a limited dispersal distance by one sex.

Wildfire has historically been the dominant force responsible for shaping numerous landscapes in both the western and boreal forests of North America. Consequently, many species are adapted to, and some are even dependent on, living in these early successional, postfire habitats. In North America, the black-backed woodpecker (*Picoides arcticus*) is perhaps the most commonly cited example of a fire specialist [14]–[17]. Black-backed woodpeckers live six to eight years, yet they only occupy fire-disturbed areas for three to five years after fire [15], [18]–[20]. Peak woodpecker densities occur two to four years following a burn [17], [19], which corresponds to high wood-boring beetle (Coeloptera: Buprestidae and Cerambycidae) densities which are their primary food source [15]. Because black-backed woodpeckers are so highly specialized to postfire habitat [16], connectivity among ‘older’ habitat patches (≈ 4 year-old) and ‘young’ (≈ 1 year-old) postfire patches may be necessary for population persistence.

While black-backed woodpeckers have been documented in unburned areas such as beetle-killed stands, nest success tends to be extremely high (80–100%) in areas that have burned at moderate to high severity [19], [20] and tends to be much lower in unburned areas (44–78%; [21]). Postfire habitats may act as source habitats [22] by increasing reproductive rates and reducing mortality rates resulting in more individuals available to contribute to immigration and emigration. In particular, juvenile survival may be markedly higher if juveniles delay dispersal and remain near their natal territory while the habitat patch has plentiful resources. Nappi and Drapeau [17] suggest that regions with high fire frequency can serve as ‘regional sources’ for areas with lower fire frequency.

In previous research, we found black-backed woodpeckers had very low genetic differentiation among patches at extremely large spatial scales (>3500 km). In addition, male and female black-backed woodpeckers likely have different dispersal behaviors in terms of crossing large inhospitable habitats such as grasslands [8]. While extremely long distance dispersal could create a pattern of low genetic differentiation [23], it is likely most birds disperse shorter distances when colonizing newly burned patches. Our goals in this study were to use molecular genetic data to assess the fine-scale genetic structure of black-backed woodpeckers, and to examine the scale at which burned habitats may serve as source habitats. Specifically, we set out to answer the following questions: 1) is there fine-scale genetic structure as a result of limited dispersal distance, 2) is the pattern of fine-scale genetic structure consistent between males and females, 3) is there a signal of increased genetic relatedness within burned patches, and 4) does genetic relatedness within burned patches increase over time?

## Methods

### Ethics Statement

All necessary permits were obtained for the described study, which complied with all relevant regulations. This work was conducted with approval from The University of Montana’s Institutional Animal Care and Use Committee, United States Fish and Wildlife Service, Montana Fish, Wildlife and Parks, Oregon Department of Fish and Wildlife, and South Dakota Department of Game, Fish, and Parks. Samples were collected on public land in the US and Canada and necessary permissions were granted from Glacier National Park, Jasper National Park, Grands Jardins Park and the US Forest Service.

### Sample Collection and Analysis

A full description of sampling protocols, DNA extraction, genotyping protocols and analyses can be found in Pierson et al. [8].

To assess fine-scale structure within and among burned areas, two field locations in western Montana (Missoula: MSLA and Glacier: GL) were identified that had three burned areas within 50 km of each other (Figure S1 in Supporting Information; BLM: Black Mountain fire, BM: Boles Meadow fire, FC: Fish Creek fire, RB: Robert fire, WC: Wedge Canyon fire, TR: Trapper fire). These areas ranged in size from approximately 4,000 to 16,000 hectares and burned in 2003. Sampling occurred between 2004 and 2007. In addition, samples were collected as part of a larger scale study in Alberta, Idaho, Oregon, South Dakota, and Eastern Canada (Figure S1 in Supporting Information; [8]). Only samples collected within five years after an area burned are included in these analyses.

Adult woodpeckers were captured at the nest site during the nestling stage to reduce the chance of nest abandonment caused by disturbance during the incubation stage. Birds were captured using either hoop nets placed over the entrance of the cavity after the adult had entered, or with mist nets targeting birds flying to the nest cavity. While both adults were sampled when possible, attempts were made to only capture one adult per day to minimize disruption of food delivery to young. Blood was collected from the brachial vein and/or 5–10 small back feathers were collected, birds were weighed and marked with a unique color-band combination and then released. Latitude and longitude of the nest locations were recorded.

Briefly, we used the following nine microsatellite loci: *C111*, *C115*, *D118*, [24]; *RCW4* (added tail), *RCW5*, (Mullins and Haig pers. comm.); *DIU3*, *DIU4*, [25]; *HrU2*, [26]; *Lox4*, [27]. Expected heterozygosity was calculated in GDA (version 1.1; [28]) and allelic richness (A_R_) and *F_IS_* were calculated in FSTAT.

### Fine-scale Genetic Structure

We performed global spatial autocorrelation analyses to test within-patch dispersal patterns [29], [30] using GenAlEx6 [31]. Global autocorrelation analysis is a multivariate approach that can detect a spatial pattern generated by multiple loci simultaneously [29]. This approach calculates a genetic autocorrelation coefficient (*r*) for a specified set of distance classes (in this case, classes of even sample sizes). Significant spatial structure is measured using both bootstrapping and permutation tests as described in Peakall et al. [32]. We used bootstrapping (n = 999) to calculate 95% error bars around the estimate of *r* and assumed class significance when the error bar did not cross zero, which is considered a conservative approach [31]. Permutation tests (n = 999) were used to calculate a 95% confidence envelope and significance was assumed when the estimate of *r* fell outside the confidence envelope around the null hypothesis of *r* = 0. Permutation tests provide a robust estimate of significance when sample sizes are small because they use the entire data set [31].

We initially conducted a global spatial autocorrelation (GSA) that included all samples across the study and divided the sample into bins of even sample sizes to determine the largest spatial scale over which genetic structure (autocorrelation between genetic and geographic distance matrices) could be detected among individuals. We then created frequency distributions of the distances among individuals within approximately 225 km of each other, the maximum distance at which structure was detected, and conducted a GSA using variable distance classes based on the peaks of these multimodal distributions [33]. We tested for sex-biased dispersal patterns by performing GSA analyses on males and females separately [34]. We then tested whether male and female dispersal patterns were significantly different by performing a heterogeneity test to determine whether their correlograms were more different than could be expected by chance [35].

We used two different approaches to assess whether family groups may be present within burned areas. Only samples from burned areas in which at least 10 individuals were sampled over the course of the study were included in these analyses. First, we employed a two-dimensional local spatial autocorrelation (2D LSA) using Genalex6 which calculates a local autocorrelation (*lr*) for each focal point and a specified subset of *n* neighboring points. We calculated a 2D LSA for the five nearest neighbors and permutation tests were used to calculate if the local autocorrelation was significantly larger than expected by chance. While multiple comparisons are involved in this type of analyses, Bonferroni corrections are not necessary because we are only looking at a small, specific subset of points [31], therefore we used a *P* = 0.05 to indicate significant *lr* values. Next, we calculated mean genetic relatedness [36] within each burned area and performed permutations (999) to determine the range of genetic relatedness that would be expected within burned areas by chance. We then calculated mean genetic relatedness among all individuals included in the analysis as a comparison of overall relatedness among individuals. These analyses were performed using Genalex6.

To assess if relatedness increased over time, as expected if juveniles remained near the natal territory for the first few years after an area burned, we calculated mean genetic relatedness within each burned patch for three consecutive years. We took a cumulative approach by first estimating mean genetic relatedness of individuals sampled the first year, then estimated mean genetic relatedness of individuals sampled in both the first and second year (year 2), and finally pooled all samples from three consecutive years (year 3) and estimated mean genetic relatedness. We then visualized these estimates to assess if there was an increase in genetic relatedness indicated over time. Due to the small number of years and sites in this study and the cumulative nature of the samples, a formal analysis such as regression or a sign test is not appropriate to test for an increase over time. However, the goal of this approach was to simply determine if a trend may exist in order to inform future research agendas.

## Results

We analyzed samples from 233 black-backed woodpeckers across North America (Table 1). The nine loci were highly variable, conformed to Hardy-Weinberg proportions, and were not in gametic disequilibrium [8]. Expected heterozygosity (H_E_) ranged from 0.48–0.61, allelic richness (A_R_) ranged from 3.76–6.67, and F_IS_ ranged from −0.107 to 0.054.

**Table 1:** The number of individuals included in global spatial autocorrelation analyses and summary statistics of genetic diversity for each location.

Location	N_female_	N_male_	H_E_	F_IS_	AR
AB	12	9	0.61	−0.029	6.67
MSLA	22	27	0.57	0.018	5.86
GL	23	24	0.57	−0.050	5.94
OR	12	17	0.57	0.054	5.33
EC	20	32	0.59	−0.014	6.09
SD	11	10	0.48	−0.107	3.76
ID	6	8	0.57	−0.065	5.73

GL: Glacier National Park, Montana; MSLA: Missoula, Montana; OR: Silver Lake, Oregon; EC: Eastern Canada, Québec Grands Jardins Park; SD: Black Hills, South Dakota; ID: central Idaho; AB: Jasper National Park, Alberta; N_female_: number of females; N_male_ : number of males;; H_E_: expected heterozygosity; F_IS_: inbreeding coefficient [45]; AR: allelic richness.

**Figure 1:**
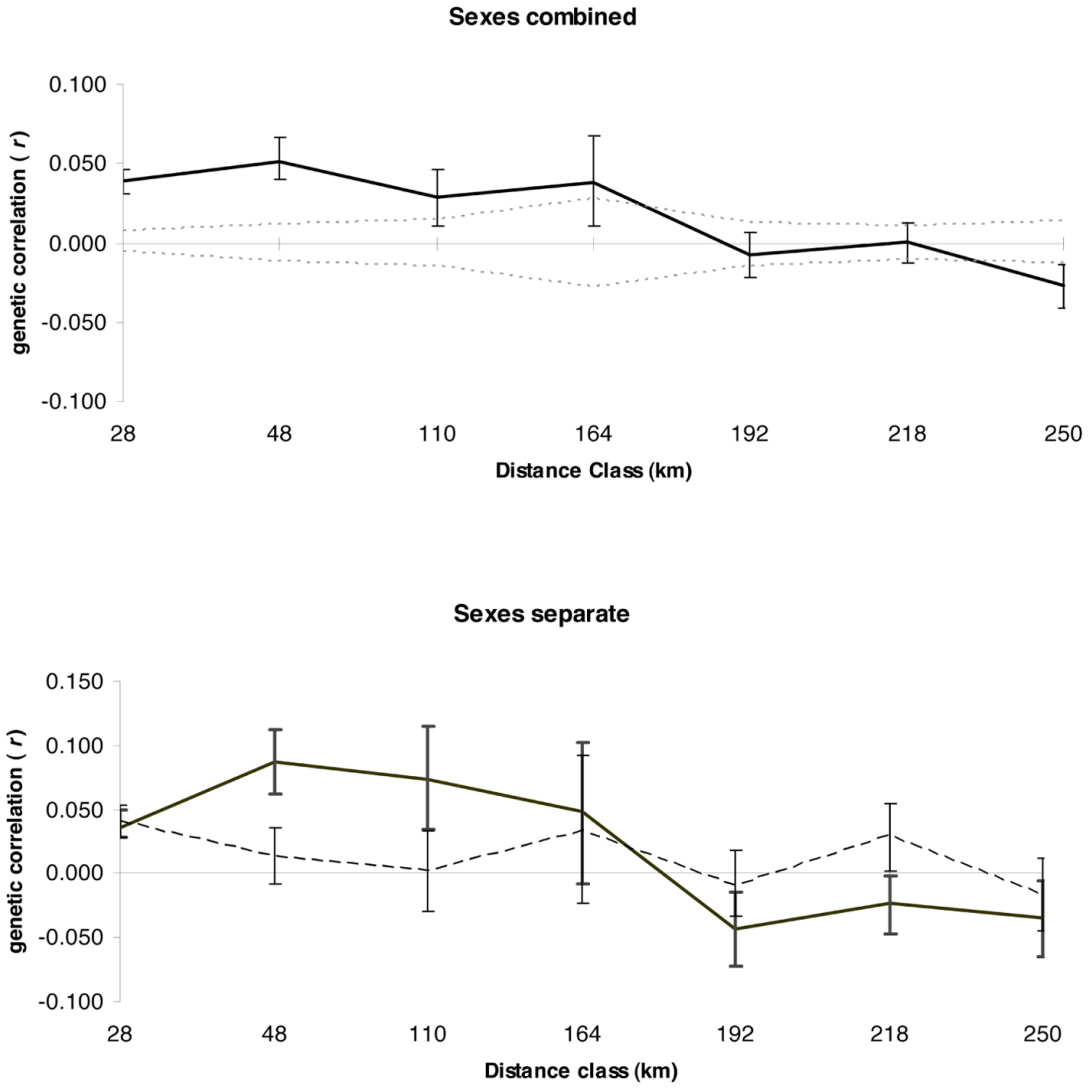
Global spatial autocorrelation at variable distance classes. Correlogram plots based on global spatial autocorrelation analyses conducted with variable distance classes. The y-axis is the genetic correlation coefficient (*r*) and the x-axis is the distance class (km). (a) both sexes combined; confidence intervals (95%) were calculated using bootstrapping (error bars) and permutation tests (dashed lines), (b) female (solid line) and male (dashed line); confidence intervals (95%) were calculated using bootstrapping (error bars).

We used global spatial autocorrelation (GSA) analysis to estimate fine-scale genetic structure. Black-backed woodpeckers displayed significantly positive genetic correlation (*r*) values in distance classes up to 164 km (Figure 1a, Table S1 in Supporting Information). We tested for sex-biased dispersal patterns by performing GSA analyses on males and females separately and performed a heterogeneity test between the correlograms to determine if they were significantly different from each other. Male and females showed significantly different patterns of genetic correlation (*P* = 0.01), with females having significantly positive *r*-values at larger distance classes than males (Figure 1b, Table S1 in Supporting Information).

**Figure 2:**
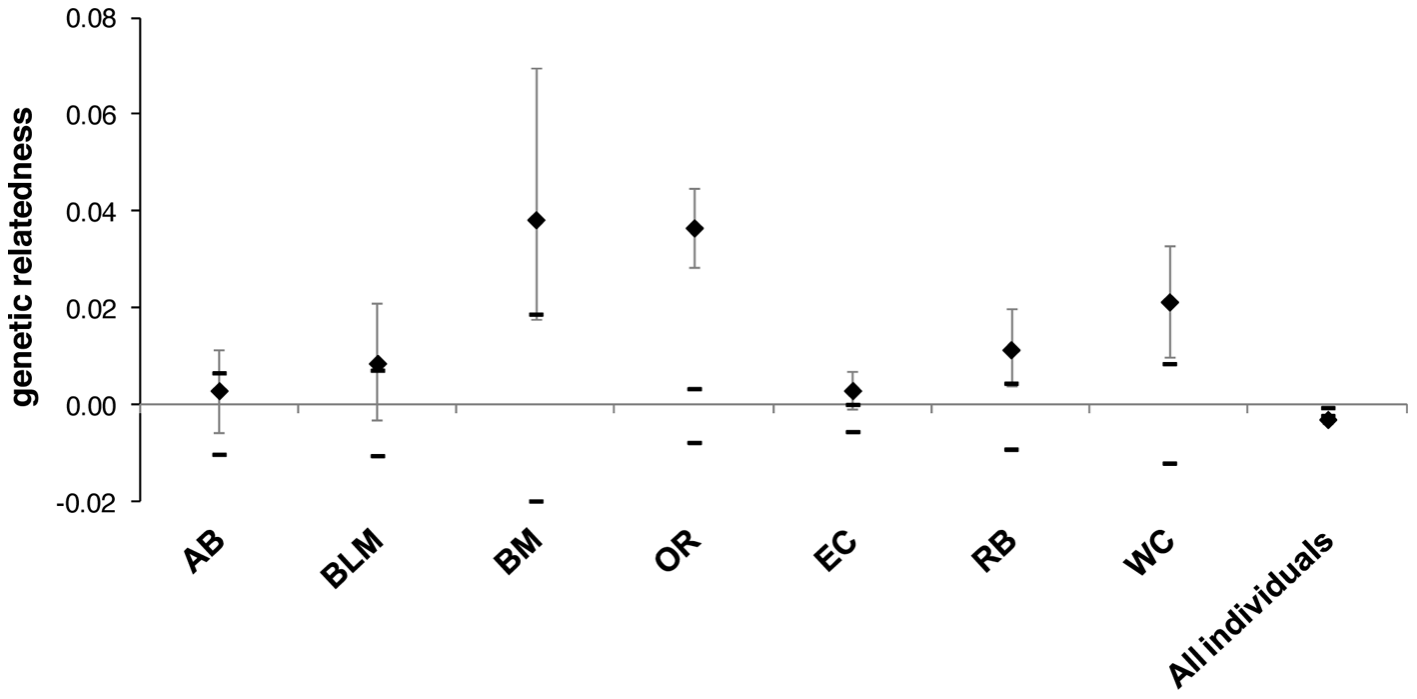
Genetic relatedness within burned patches. Estimates of mean genetic relatedness within each burned area with at least 10 individuals sampled (see Table 2 for location descriptions) and mean relatedness for all individuals combined. The 95% confidence intervals are based on both bootstrapping (error bars surrounding estimates) and permutations (error bars surrounding null expectation of 0).

**Figure 3:**
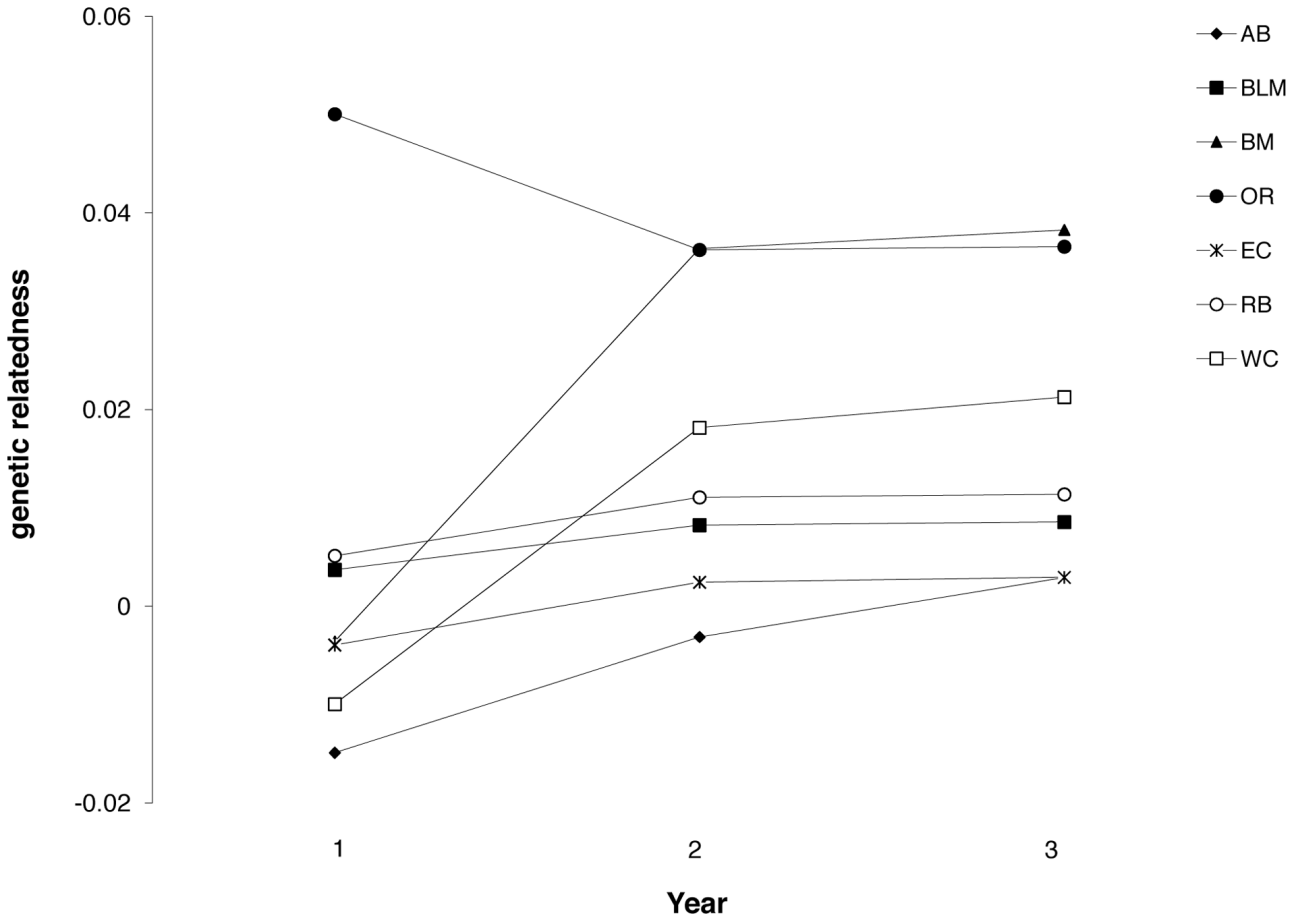
Temporal trends in genetic relatedness. Estimates of mean genetic relatedness within each burned area during the first, second and third year of sampling. Second and third years represent cumulative estimates of relatedness, that is, samples in year two include samples from both year one and year two.

We performed 2D local spatial autocorrelation (LSA) on samples in eight burned areas. All of the burned areas contained at least one individual with significantly positive *lr*-values based on a one-tailed test and higher than expected clusters of relatedness (Table 2), and most of the sites contained three or more individuals with significantly positive *lr*-values. We calculated mean genetic relatedness in seven burned areas; ID was not included as samples were not collected in multiple years. Mean relatedness was negative when calculated for all individuals in this analysis (−0.003) and values tended to be small but significant within burned areas (0.003–0.038; Figure 2). Most sites (five of seven) had a mean relatedness larger than would be expected by chance and all sites had a larger genetic relatedness than mean relatedness overall (Figure 2). Additionally, genetic relatedness increased over time in six of the seven burned areas (Figure 3).

**Table 2:** The number of individuals included (N) in 2D LSA for each burned area: AB: Jasper National Park, Alberta; BLM: Black Mountain fire near Missoula, MT; BM: Boles Meadow fire near Missoula, MT; RB: Robert fire within Glacier National Park, MT; WC: Wedge Canyon fire within Glacier National Park, MT; ID: central Idaho; OR: Silver Lake, Oregon; EC: Eastern Canada, Québec Grands Jardins Park; the percent of individuals that had significant genetic clusters surrounding them, including the range of significance values and local genetic autocorrelation values (*lr*).

Location	N	N significant	% significant	P-value range	*lr* range
AB	21	3	14	0.005–0.011	0.20–0.24
BLM	21	5	24	0.003–0.026	0.17–0.25
BM	11	5	45	0.003–0.045	0.15–0.24
OR	29	4	14	0.002–0.02	0.17–0.26
EC	49	5	10	0.003–0.041	0.14–0.27
RB	24	1	4	0.018	0.18
WC	16	3	19	0.004–0.032	0.16–0.25
ID	10	1	10	0.041	0.14

## Discussion

**Figure 4:**
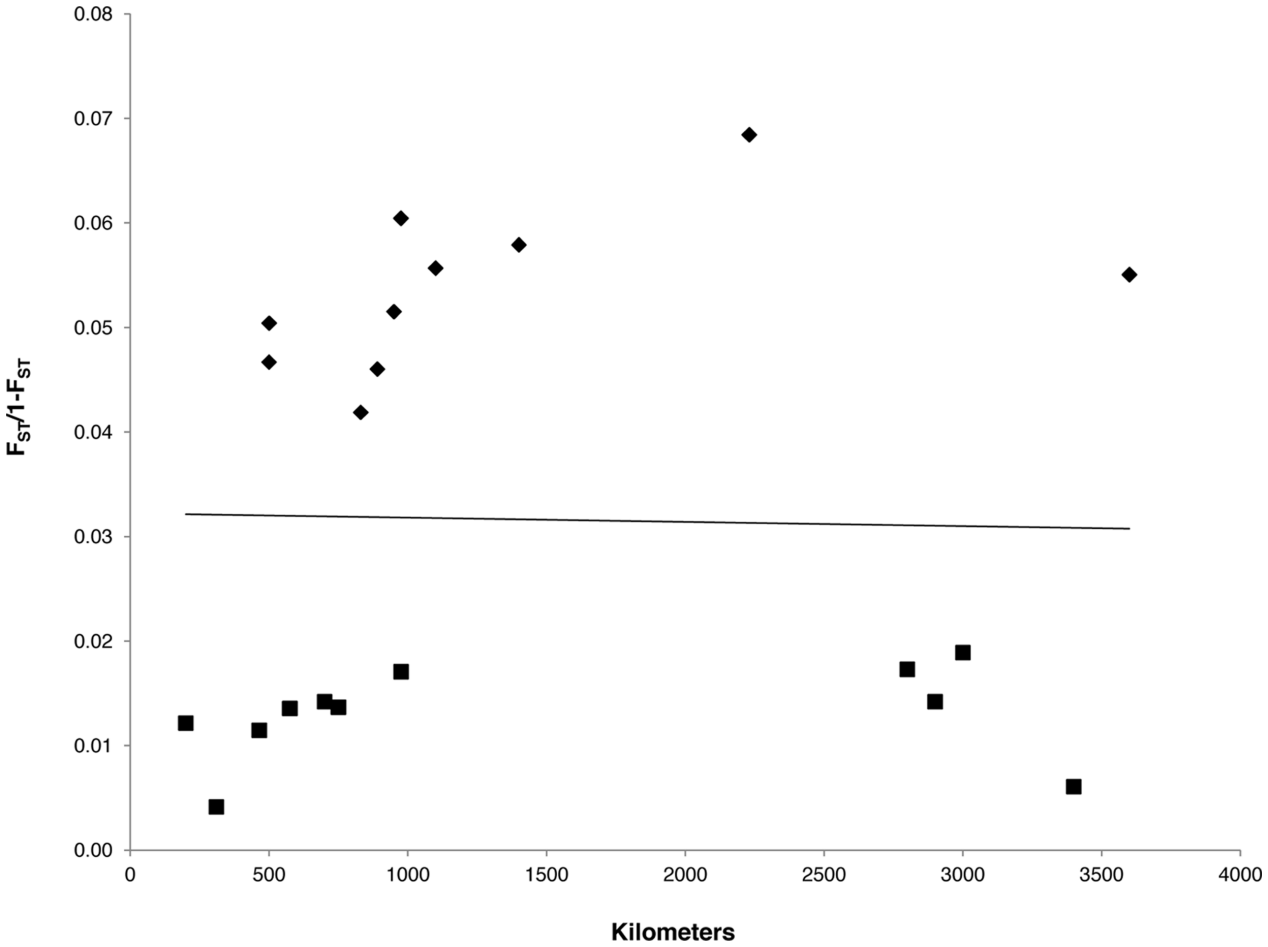
Large-scale genetic structure among sampling locations. A scatterplot of genetic distance versus geographic distance with a regression line showing the lack of a correlation between the two parameters (*P* = 0.30; Mantel test). Genetic distance was based on pairwise estimates of F_ST_/1- F_ST_ among all seven sampling locations: Alberta (AB), Missoula (MSLA), Glacier (GL), Oregon (OR), Eastern Canada (EC), South Dakota (SD), and Idaho (ID). Geographic distances were calculated as the straight-line distance from the center of each sampling location. Pairwise estimates between locations within the boreal forest (AB, MSLA, GL, EC, ID) are represented with squares; pairwise estimates involving at least one location outside the boreal forest (OR, SD) are represented with diamonds.

Our goal was to test if a disturbance-dependent specialist can accrue significant fine-scale genetic structure in the limited period of time that their natal habitat persists, despite displaying near panmixia over extremely large spatial scales. Frequent colonization events combined with a high rate of population turnover usually leads to a lack of genetic structure among subpopulations [37]. In a concurrent study, we found a lack of large-scale genetic structure in black-backed woodpeckers at extremely large spatial scales [8]. Specifically, we did not detect any signal of isolation by distance across their entire range (Figure 4). Genetic differentiation among sites within the boreal forest population was negligible (F_ST_ <0.02, Figure 4) even at maximal distances (3500 km+) indicating the boreal forest population is essentially panmictic [38], [8]. Yet we detected a signal of fine-scale genetic structure based on both estimates of spatial autocorrelation and genetic relatedness despite the highly ephemeral nature of the habitat patches these woodpeckers were occupying. Fine-scale genetic structure due to an individual’s limited dispersal distance can exist despite high gene flow across large spatial scales [39]. However, limited dispersal usually results in a signal of isolation by distance at larger spatial scales [40]. This discrepancy is likely due to frequent colonization of patches by birds from multiple source populations causing a mixing of genotypes over time resulting in a similarity in allele frequencies [41]. The individual-based analyses presented in this study were able to tease out temporal patterns in fine-scale genetic structure over a short time scale resulting from individual behavior.

### What can fine-scale Structure tell us about Avian Dispersal Behavior?

A significant positive genetic correlation among individuals is expected when dispersal is limited [12], [30]. Peakall et al. [32] found that the scale at which positive genetic correlation persists in bush rats matched demographic data on dispersal distance. In birds, two studies have evaluated the usefulness of spatial autocorrelation techniques in assessing dispersal patterns by comparing demographic data to correlograms based on individually based genetic data [30], [42]. These studies found a high level of concurrence between data sets. Black-backed woodpeckers displayed positive genetic structure at a fairly large spatial scale (164 km); however this distance is not nearly far enough to create the panmictic genetic signature observed across the boreal forest (Figure 4).

The presence of genetic structure at this finer scale suggests that a mechanism other than long-distance dispersal is responsible for the lack of structure at the larger scale and may provide general information on the spatial scale of the genetic neighborhood [12]. On average, the scale of the genetic neighborhood for black-backed woodpeckers appears to be between 164–192 km, a pattern likely driven mainly by limited dispersal in females. Black-backed woodpeckers likely disperse multiple times during their lifespan as burned patches are optimal habitat for three to five years and these woodpeckers can live at least eight years [15]. The fine-scale structure detected by genetic autocorrelation is likely due to individual bird’s behavior when dispersing to exploit newly created habitat patches, and therefore it is not surprising to detect ‘fine-scale’ spatial structure at a somewhat larger scale than small passerines such as fairy wrens [30]. However, given the dynamic nature of fire regimes, a genetic neighborhood of only 164 km suggests female birds, on average, locate optimal habitat within fairly close proximity to where they were born as opposed to dispersing unlimited distances to locate optimal habitat. This has implications for the management of burned forests as managers need to be aware of the spatial and temporal context of habitat when making decisions regarding salvage logging and prescribed burning. This will become an increasingly important and difficult challenge as land use changes, such as timber harvest and fire suppression, combine with the effects of climate change.

When sex-biased dispersal is present, fine-scale structure may be due to restricted dispersal in only one sex even though fine-scale structure is observed when the sexes are pooled. When examined alone, only the sex with restricted dispersal will display fine-scale structure and the sex that disperses will typically show either a lack of fine-scale structure or markedly less fine-scale structure relative to the sex with restricted dispersal [34], [42]. Black-backed woodpeckers had a clear signal of male-biased dispersal. When females are examined alone, there was a positive genetic correlation up to 48–110 km indicating positive genetic structure at a spatial scale greater than the burned patches occupied by birds. Males had a positive genetic correlation in the smallest distance class only, which reflects patterns within burned patches. This signal is likely due to delayed dispersal of juvenile males as opposed to differences in dispersal distance once birds leave their initial natal burned patch to colonize a newly burned area.

Male-biased dispersal in black-backed woodpeckers is concurrent with the differences in behavior detected at the large scale, where female black-backed woodpeckers appeared to respond differently to crossing gaps outside the boreal forest [8]. Sex-biased dispersal has been proposed as a mechanism to avoid inbreeding and is usually female-biased in birds [13]. Both sexes must frequently colonize new habitat patches given the short duration that burned patches are optimal habitat, therefore it is surprising to find sex-biased dispersal at all. Despite the relatively large scale of the genetic neighborhood (≈ 150 km), there may be very few recently burned patches of forest within that distance from an aging patch thereby retaining the need for sex-biased dispersal as an inbreeding avoidance mechanism. Additionally, males may benefit from exploring larger distances and encountering more potential habitat patches which may result in acquiring higher quality territories.

### Can Fine-scale Genetic Structure Inform Population (or Source) Dynamics?

We wanted to test whether delayed juvenile dispersal potentially accounts for the fine-scale genetic signature. Nappi and Drapeau [17] suggest that regions with high fire frequency can serve as ‘regional sources’ for areas with lower fire frequency. In particular, juvenile survival may be markedly higher if juveniles delay dispersal and remain near their natal territory while the habitat patch has plentiful resources. We predicted that juveniles would delay dispersal to exploit habitat that is high in food and nesting resources and thus we would see genetic clusters within burned areas as evidenced by the 2D LSA analysis, and an increase of genetic relatedness (*r*) compared to the population overall that appears to increase through time. This prediction was largely supported with all eight burned areas showing a relatively large number of genetic clusters with strong signals of genetic correlation (Table 2), and patterns of genetic relatedness that were higher within burned patches compared to the average among all individuals (Figure 2) and a strong trend of increased relatedness over time (Figure 3). The majority of the increase in genetic relatedness appeared to occur between the first and second year of sampling with smaller increases observed when a third year of samples was added to the analysis. This signal could be due to unrelated birds colonizing areas so a marked increase occurs when related juveniles remain in the burned patch and the addition of more related juveniles in third year results in a comparatively smaller increase in relatedness.

Although this is a short time scale to assess changes in structure, Nussey et al. [43] found spatial genetic structure can change rapidly through time as a result of changes in population size and decreasing polygyny in red deer (*Cervus elaphus*). Black-backed woodpeckers did show evidence of increased genetic relatedness over time in 86% of the burned areas sampled (Figure 3). The higher than expected genetic relatedness within patches that appears to accumulate temporally supports the hypothesis that juveniles stay near their natal territory while postfire habitat has plentiful resources [17], [18], [22]. Additional anecdotal support includes a black-backed woodpecker banded as a nestling that was documented breeding in the same wildfire the following season. It appears juvenile dispersal may be delayed, which likely increases juvenile survival, and helps to explain how burned areas act as source habitat. These results highlight the importance of temporal change in genetic structure over short time periods [43] and illustrate how temporal variation in genetic structure can provide insights into demographic processes.

Early postfire habitat may provide source habitat for some woodpecker species because abundance is higher in burned areas versus unburned areas for species that occupy both habitat types [22] and may provide emigrants. We found female black-backed woodpeckers likely disperse less than 110 km, providing the first details on the spatial scale that these burned areas may act as a source, which is well above the average distance between the closest fire events (38.5 km) happening within successive years in the eastern boreal forest of Canada [44]. Hence, in this part of its range, the long-term persistence of black-backed woodpeckers in the landscape is likely ensured by the connectivity of wildfires.

Disturbance-dependent species have evolved with a natural mosaic of shifting habitat patches. The dynamic nature of ephemeral habitats makes it challenging to understand habitat connectivity and resulting metapopulation dynamics. Our results indicate that disturbance-dependent species that outlive their ephemeral patches can accrue significant fine-scale spatial structure despite a lack of genetic structure at larger spatial scales.

## Supporting Information

Figure S1 **A map of the United States and Canada showing the hierarchical sampling design including (a) the location of the seven broad-scale study sites: AB: Jasper National Park, Alberta; MSLA: Missoula, Montana; GL: Glacier National Park, Montana; OR: Silver Lake, Oregon; EC: Eastern Canada, Québec Grands Jardins Park; SD: Black Hills, South Dakota; ID: central Idaho; and (b) the two study sites within western Montana that each have three areas that burned in 2003: Missoula – BLM: Black Mountain fire; BM: Boles Meadow fire; FC: Fish Creek fire and Glacier National Park – WC: Wedge Canyon fire; RB: Robert fire; TR: Trapper fire.**
(TIF)

Table S1 **Results from a one-tailed test for positive genetic autocorrelation (** ***r*** **), which is expected when there is limited dispersal, for both sexes, then for males and females separately.** The number of pairwise comparisons (N) per distance class (km), estimated genetic correlation for each distance class (r), and the probability the estimated *r* is greater than expected based on 1000 permutations (P); significant values are indicated in bold.
(DOCX)
