# Supplementary figures and images for: Breed Locally, Disperse Globally: Fine-Scale Genetic Structure Despite Landscape-Scale Panmixia in a Fire-Specialist
Source: PLoS One. 2013 Jun 25;8(6):e67248. doi: 10.1371/journal.pone.0067248 (PMC3692495; doi:10.1371/journal.pone.0067248)

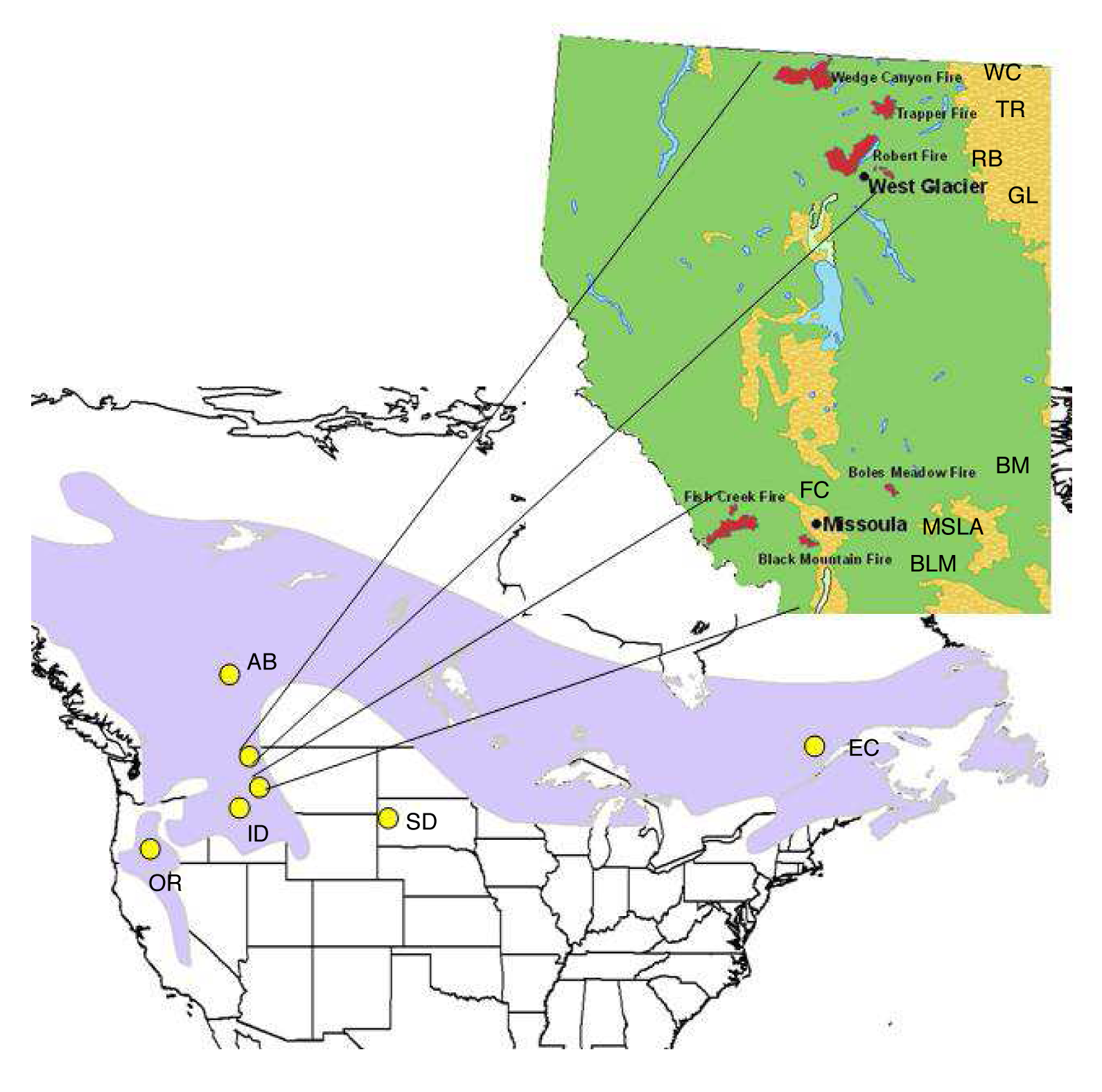

Supplement: Figure S1 — A map of the United States and Canada showing the hierarchical sampling design including (a) the location of the seven broad-scale study sites: AB: Jasper National Park, Alberta; MSLA: Missoula, Montana; GL: Glacier National Park, Montana; OR: Silver Lake, Oregon; EC: Eastern Canada, Québec Grands Jardins Park; SD: Black Hills, South Dakota; ID: central Idaho; and (b) the two study sites within western Montana that each have three areas that burned in 2003: Missoula – BLM: Black Mountain fire; BM: Boles Meadow fire; FC: Fish Creek fire and Glacier National Park – WC: Wedge Canyon fire; RB: Robert fire; TR: Trapper fire. (TIF) [file pone.0067248.s001.tif]
