# Supplementary material for: Breed Locally, Disperse Globally: Fine-Scale Genetic Structure Despite Landscape-Scale Panmixia in a Fire-Specialist
Source: PLoS One. 2013 Jun 25;8(6):e67248. doi: 10.1371/journal.pone.0067248 (PMC3692495; doi:10.1371/journal.pone.0067248)
Supplement: Table S1 — Results from a one-tailed test for positive genetic autocorrelation ( r ), which is expected when there is limited dispersal, for both sexes, then for males and females separately. The number of pairwise comparisons (N) per distance class (km), estimated genetic correlation for each distance class (r), and the probability the estimated r is greater than expected based on 1000 permutations (P); significant values are indicated in bold. (DOCX) [file pone.0067248.s002.docx]

Table S1. Results from a one-tailed test for positive genetic autocorrelation (*r*) which is expected when there is limited dispersal including both sexes, then for males and females separately. The number of pairwise comparisons (N) per distance class (km), estimated genetic correlation for each distance class (r), and the probability the estimated *r* is greater than expected based on 1000 permutations (P); significant values are indicated in bold

|  | **Distance class (km)** | **28** | **48** | **110** | **164** | **192** | **218** | **250** |
| --- | --- | --- | --- | --- | --- | --- | --- | --- |
| BOTH | **N** | 2895 | 960 | 575 | 159 | 624 | 975 | 647 |
|  | **r** | 0.039 | 0.051 | 0.029 | 0.038 | -0.008 | 0.001 | -0.027 |
|  | ***P*** | 0.000 | 0.000 | 0.000 | 0.006 | 0.869 | 0.468 | 1.000 |
| MALE | **N** | 935 | 259 | 155 | 47 | 191 | 272 | 171 |
|  | **r** | 0.041 | 0.013 | 0.002 | 0.033 | -0.009 | 0.029 | -0.018 |
|  | ***P*** | 0.001 | 0.132 | 0.457 | 0.098 | 0.785 | 0.003 | 0.891 |
| FEMALE | **N** | 512 | 221 | 127 | 33 | 124 | 215 | 151 |
|  | **r** | 0.036 | 0.087 | 0.074 | 0.048 | -0.043 | -0.023 | -0.035 |
|  | ***P*** | 0.001 | 0.001 | 0.001 | 0.069 | 0.996 | 0.974 | 0.993 |
